# Physiological Notes on Primary Education and the Study of Language

**Published:** 1889-08

**Authors:** 


**Physiological Notes on Primary Education and the Study of Language.** By MARY PUTNAM JACOBI, M. D. 12mo, pp. 120. The Knickerbocker Press of G. P. Putnam's Sons. 1889.

The system of education here outlined is a most perfect one. It overthrows the time-honored method of beginning with reading, writing, and arithmetic, in which, as the author says, there is an exquisite absurdity. The author further says: "The first intellectual faculties to be trained are perception and memory. The subjects of the child's first studies should, therefore, be selected not on account of their ultimate utility, but on account of these faculties. What sense is there, then, in beginning education with instruction in the arts of reading and writing?" Almost every one can look back and remember tears shed in the mastering of abstract propositions, which they afterwards recited, parrot-like, without understanding. This is a book that should be read by all teachers of the young, in the hope that it may lead to a better order of things.

There is only one exception to be taken to this admirable work. The child's education should *not* begin at the age of four. With very few exceptions, the physical force would soon be exhausted, leaving no endurance for the years when it is most needed. There is something pathetic in a child of five and a half being able to dictate the following to be written in a journal: "The episperm, on the under surface of Tertius, is all black, and has split, leaving a space the shape of an equilateral triangle, with the apex pointing to the convex edge of the cotyledons." The last half of the book contains many valuable suggestions in regard to the study of language. H. B.

